# Low Seroprevalence of SARS-CoV-2 Antibodies during Systematic Antibody Screening and Serum Responses in Patients after COVID-19 in a German Transplant Center

**DOI:** 10.3390/jcm9113401

**Published:** 2020-10-23

**Authors:** Mira Choi, Friederike Bachmann, Marcel Ganesh Naik, Wiebke Duettmann, Michael Duerr, Bianca Zukunft, Tatjana Schwarz, Victor Max Corman, Lutz Liefeldt, Klemens Budde, Fabian Halleck

**Affiliations:** 1 Department of Nephrology and Medical Intensive Care, Charité- Universitätsmedizin Berlin, 13353 Berlin, Germany; friederike.bachmann@charite.de (F.B.); marcel.naik@charite.de (M.G.N.); wiebke.duettmann@charite.de (W.D.); michael.duerr@charite.de (M.D.); bianca.zukunft@charite.de (B.Z.); lutz.liefeldt@charite.de (L.L.); klemens.budde@charite.de (K.B.); fabian.halleck@charite.de (F.H.); 2 Institute of Virology, Charité-Universitätsmedizin Berlin, German Center for Infection Research (DZIF), and Berlin Institute of Health, 10117 Berlin, Germany; tatjana.schwarz@charite.de (T.S.); victor.corman@charite.de (V.M.C.)

**Keywords:** COVID-19, kidney transplantation, SARS-CoV-2 serology, antibody testing

## Abstract

The coronavirus disease 2019 (COVID-19) pandemic caused by SARS-CoV-2 denotes a global health issue. Data regarding COVID-19 incidence in kidney transplant recipients (KTR) are sparse. From 19 March to 19 May 2020, we performed a systematic screening for COVID-19 in KTR. Tests included serum analysis for SARS-CoV-2 antibodies using S protein-based immunofluorescence, anti-SARS-CoV-2 S1 immunoglobulin G (IgG) and immunoglobulin A (IgA) enzyme-linked immunosorbent assays (ELISA), and/or quantitative reverse transcription polymerase chain reaction (qRT-PCR) from nasal-throat swabs. Outpatient serum samples from KTR with PCR confirmed COVID-19, and swab samples from recipients (+donors) undergoing kidney transplantation were analyzed. Out of 223 samples from outpatients, 13 patients were positive with solely anti-SARS-CoV-2-IgA and 3 with both anti-IgA and anti-IgG. In total, 53 patients were symptomatic in the past, but positive results could be found in both symptomatic and asymptomatic patients. After an in depth analysis using immunofluorescence and neutralization tests in 2 KTR, recent COVID-19 infection remained highly suspicious. Apart from outpatient visits, only 5 out of 2044 KTR were symptomatic and tested positive via PCR, of which 4 recovered and one died. All patients showed seroconversion during the course of the disease. This study demonstrated a low seroprevalence in a German KTR cohort, and seroconversion of IgA and IgG after COVID-19 could be demonstrated. Effective containment strategies enabled us to continue our transplant program.

## 1. Introduction

The current coronavirus disease 2019 (COVID-19) pandemic is caused by a novel coronavirus named severe acute respiratory syndrome coronavirus 2 (SARS-CoV-2) and has become a worldwide global health issue with rates of infection still increasing. Although older patients and those with underlying chronic diseases are at risk for the worst outcomes [1], incidence and clinical data in immunosuppressed patients are sparse.

In general, a case fatality rate of 6.8% among 46,959 COVID-19 patients was described in a recent metanalysis [2], of which 29.3% required a stay in an intensive care unit and 28.8% experienced acute respiratory distress syndrome. In a larger case series of 90 organ transplant recipients, including 43 patients after renal transplantation with COVID-19 from an outbreak in New York, high rates of severe disease (39%) and mortality (24%) were observed [3]. However, single- and multi- center studies reported similar outcomes from organ transplant patients after COVID-19, mainly without increased mortality and morbidity compared to the general population [4,5,6,7].

On January 27th, the first case of COVID-19 was diagnosed in Berlin. On March 9th, 1000 cases were registered in Germany, leading to a nationwide shutdown on March 27th following recommendations from the Robert Koch Institute. While transplant centers in neighboring countries interrupted their transplant activities, the German Transplant Society reported ongoing activities in all centers. Regarding diagnostic strategies to confirm COVID-19 RNA, reverse transcription polymerase chain reaction (RT-PCR) test swab samples are routinely used to detect SARS-CoV-2 infections, but with limited sensitivity [8,9]. Positive test results are strongly restricted to the acute phase of infection and previous infections might be left undetected. The host immune response to COVID-19 is characterized by the development of SARS-CoV-2 antibodies around the second week after infection [10]. Therefore, the application of serological testing has become an important diagnostic tool [10,11], especially in patients with mild to moderate disease severity or who present late and might have been missed. Although case series have been reported to have SARS-CoV-2 serologic responses in solid organ recipients (SOT) after COVID-19 [12,13], little is known about the seroprevalence and serological responses in renal transplant patients with COVID-19. SARS-CoV-2 target antigens were used for recent antibody assays including two of the four major structural proteins of coronavirus: the spike surface glycoprotein (S) or the nucleocapsid (N). From current immunoassays, the Euroimmun enzyme-linked immunosorbent assay (ELISA) has been established at our hospital and recently received emergency use authorization from the Food and Drug Administration (FDA).

The aim of our study was to systematically screen patients after renal or combined renal transplantation to determine the prevalence of clinically mild or inapparent SARS-CoV-2 infection using serological assays in our outpatient section. To compare serological findings, we report clinical courses and serological responses of renal transplant patients with confirmed COVID-19 infection at the Charité, Berlin, Germany.

## 2. Experimental Section

### 2.1. Study Design

From 19 March until 19 May 2020, serum samples from 223 renal and combined-renal transplant patients in the outpatient department at the Charité were assessed routinely for serological screening for SARS-CoV-2. Due to hygienic precautions, patients were given advice to come to our outpatient transplant center only if clinically stable without COVID-19 symptoms. In the case of severe COVID-19 symptoms, patients were directly referred to our emergency department for further diagnostics in a separate unit. All patients were contacted by telephone shortly before their scheduled outpatient visits, wherein a short history was taken and patients were counseled. If necessary, a personal visit or a video consultation was scheduled. All patients were adults (>18 years). Patients were asked for recent or previous symptoms at the time of antibody testing.

To monitor serum responses in patients with a polymerase chain reaction (PCR)-confirmed diagnosis of COVID-19, serum samples were obtained during the course of the disease and/or follow up visits after recovery. To minimize the risk of infection, patients with end-stage renal disease undergoing renal transplantation during our observation period were screened for clinically inapparent SARS-CoV-2 infection using throat-nasal swabs. In the case of living donors, both the donor and recipient were tested.

### 2.2. Subjects

This study was approved by the local institutional review board of the ethics committee of Charité Universitätsmedizin Berlin, Germany (approval number EA1/252/17). All patients gave their written informed consent.

### 2.3. Serological Testing for SARS-CoV-2 Antibodies

To test for SARS-CoV-2 specific antibodies, we performed anti-SARS-CoV-2 S1 IgG and IgA ELISAs according to the manufacturer’s instructions (Euroimmun Medizinische Labordiagnostika AG, Lübeck, Germany). Serum samples were analyzed at a 1:101 dilution using the fully automated Euroimmun Analyzer I (Euroimmun Medizinische Labordiagnostika AG). Optical density ratios of 0.8–1.09 were considered borderline positive, and above 1.1 reactive for IgG and IgA. In patients with COVID-19, an anti-SARS-CoV-2 IgG ELISA against the nucleocapsid (N) protein was performed (Euroimmun Medizinische Labordiagnostika AG).

To confirm ELISA, reactive sera borderline and positive samples were analyzed using a recombinant SARS-CoV-2 S protein-based immunofluorescence test (IFT), as has been described previously [10]. Briefly, VeroB4 cells expressing the spike protein of SARS-CoV-2 were used to detect the presence of IgG antibodies. For the screening serum, samples were diluted 1:10 and 1:100. Finally, in-house plaque reduction neutralization test (PRNT) was performed for ELISA and IFT positive samples, as has been described previously [10].

### 2.4. RT-PCR Methodology for SARS-CoV-2 Infection

Throat and nasal swabs were examined by RT-PCR using targets in the E- and RdRp genes, as has been described previously [14]. Primer and probes were synthesized and provided by Tib-Molbiol (Berlin, Germany), and samples were tested for SARS-CoV-2 RNA using the Roche MagNApure 96 and Roche Light Cycler 480II systems (Roche, Penzberg, Germany).

### 2.5. Statistics

Values are given as medians with an interquartile range, unless stated otherwise. IBM® SPSS® statistics version 25.0 (IBM Deutschland, Ehningen, Germany) was used for statistical analysis. Group differences were assessed in a univariate analysis using a Chi square test. *p* values of < 0.05 were considered statistically significant.

## 3. Results

### 3.1. Patient Characteristics and SARS-CoV-2 Seroprevalence in Patients after Renal Transplantation in Our Outpatient Department

**Figure 1:**
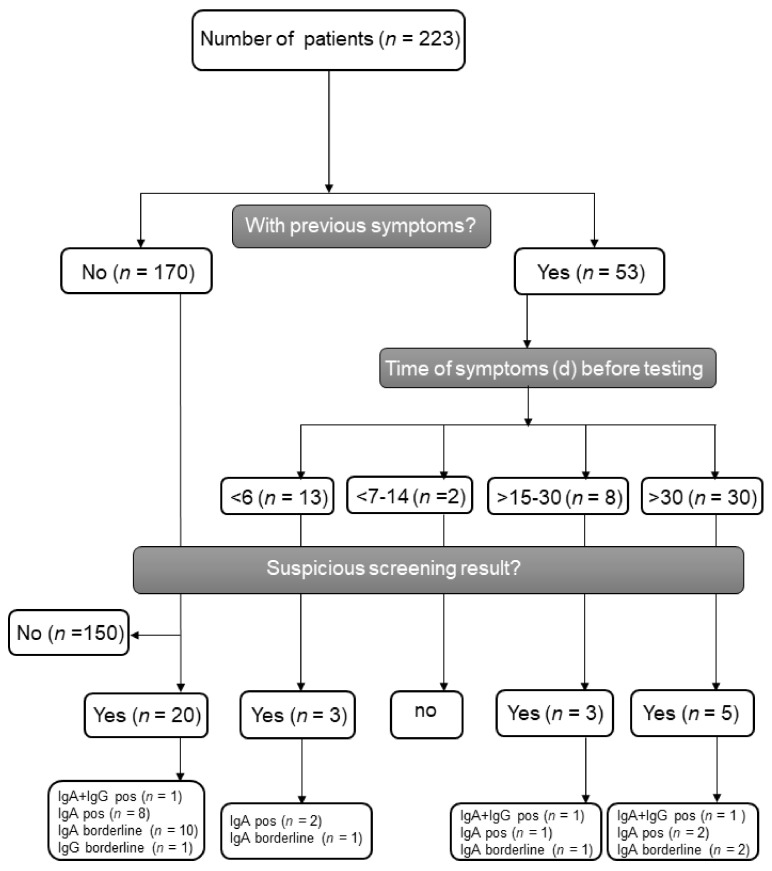
Flow scheme of SARS-CoV-2 antibody screening and test results divided by previous symptoms. IgA = Immunoglobulin A, IgG = Immunoglobulin G, pos = positive.

**Figure 2:**
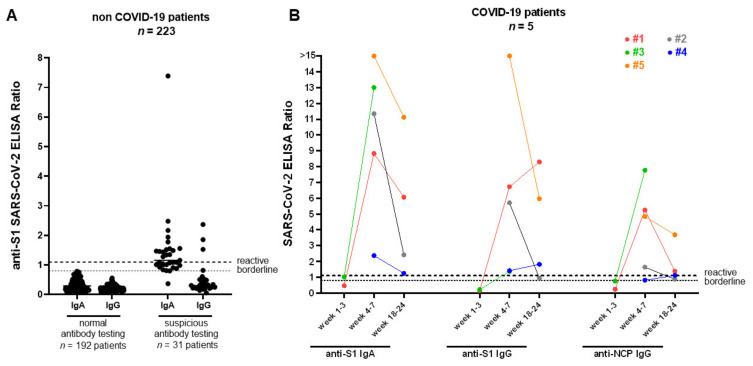
SARS-CoV-2 IgA and IgG responses for non-COVID-19 and COVID-19 patients in the kidney transplant recipients (KTR) cohort. (**A**) Spike (S1) IgA and IgG antibodies from 223 non-COVID-19 (black dots) patients were measured using a commercial ELISA. Of the non-COVID-19 patients, 192 were IgA and IgG negative and 31 patients showed borderline/reactive results. (**B**) anti-SARS-CoV-2 S1 IgG, anti-SARS-CoV-2 S1 IgA, and anti-SARS-CoV-2 NCP (nucleocapsid) IgG from 5 PCR-confirmed COVID-19 patients (colored dots) were detected at 1–3 weeks (case 1 and 3), 4–7 weeks, and 18–24 weeks (cases 1, 2, 4, and 5) after symptoms onset, respectively (see also supplementary Table S1 for further details regarding patients characteristics). All COVID-19 patients underwent a seroconversion 4 to 7 weeks after symptom onset. SARS-CoV-2 = severe acute respiratory syndrome coronavirus 2, IgA = Immunoglobulin A, IgG = Immunoglobulin G, COVID-19 = Coronavirus Disease 2019, ELISA = enzyme-linked immunosorbent assay, PCR = polymerase chain reaction.

Between 19 March and 19 May 2020, 223 patients were examined for possible active or previous SARS-CoV-2 infection. We obtained serum samples during regular outpatient visits; from among the study sample, 53 patients had been symptomatic in the past with ear-nose-throat and/or pulmonary infections (n = 44), diarrhea (n = 3), or other unspecific complaints (n = 6). Patient demographics are depicted in Table 1. Recipients had a median age of 46 years (interquartile range (IQR) 34.56) at the time of transplantation. The median age at assessment was 54 years (IQR 42.64) and the median time after transplantation was 66 months (IQR 14.155), respectively. The proportion of males was slightly higher (61.9%) than females. In total, 210 patients had undergone kidney transplantation, among which nine had undergone combined kidney–pancreas transplantation, three had undergone combined kidney–liver transplantation, and one was a multivisceral transplant. The median time between the date of previous symptoms and date of serum sampling was 46 days (IQR, 31.60). Furthermore, 13 (5.8%) patients showed mild symptoms (*n* = 10 respiratory, *n* = 3 gastrointestinal) at the time of assessment. By using ELISA antibody testing, 13 patients (5.8%) showed a solely positive SARS-CoV-2-IgA antibody result and three (1.3%) a combined positive IgA and IgG antibody result (median ratio IgA 1.49, range 1.28–7.39, median ratio IgG 1.86, range 1.53–2.37). The distribution of positive serologic findings according to the presence of previous symptoms before the serum assessment are depicted in Figure 1, demonstrating a detection of antibodies in both symptomatic and asymptomatic patients. Another 15 patients (6.7%) showed borderline results regarding anti-IgA (N = 14) and anti-IgG (N = 1) antibodies (Figure 2A). To further analyze positive findings for patients with borderline or reactive ELISA results, recombinant SARS CoV-2 S protein-based IFT were performed. Only 2 of 16 patients remained highly suspicious for recent COVID-19 infection. Both patients had reactive IgA and IgG antibody results, yet both turned out negative in the plaque reduction neutralization test (PRNT). Interestingly, both patients had symptoms of cough, which was compatible with a SARS-CoV-2 infection or infection with another respiratory virus (e.g., endemic CoV) 3 and 12 weeks before retrieving serum samples, respectively. Follow up sera after 24 and 55 days revealed positive IgA findings in both, as well as one positive IgG and one borderline IgG result. Notably, three other patients reported contact with confirmed COVID-19 positive persons, but none showed reactive IgA or IgG antibody results.

Patients undergoing renal transplantation during our observation period (19 deceased donor–recipients, four live donor–recipient pairs) were tested for SARS-CoV-2 prior to transplantation and turned out to be negative. All patients and donors were asymptomatic at that time.

### 3.2. Seroconversion in Renal Transplant Patients after SARS-CoV-2 Infection

Until the end of our observation period, outside of the outpatient section, 5 out of 2044 KTR from our center were symptomatic and tested positive with PCR. Symptoms were fever (*n* = 5), malaise (*n* = 5), coughing (*n* = 4), dyspnea (*n* = 3) and diarrhea (*n* = 1).

Immunosuppression was reduced in three patients as follows: (1) from baseline cyclosporine (CsA), mycophenolic acid (MPA) and steroid (ST) to CsA and ST; (2) from baseline tacrolimus (TAC), MPA, ST to TAC and ST; (3) from TAC, MPA and ST to steroid monotherapy in a patient with severe respiratory distress syndrome requiring mechanical ventilation. This last patient deteriorated rapidly and died 24 days after admission. In two patients with mild symptoms, dual immunosuppression (TAC, MPA) was not altered.

Thus, four out of five patients recovered and one patient died due to severe COVID-19 pneumonia. Baseline characteristics of COVID-19 PCR and SARS-CoV-2 antibody results are depicted in Table 2 and Figure 2B, respectively. All except one patient were hospitalized. During the course of disease, all patients showed seroconversion for IgA and IgG (median time between start of symptoms and serum assessment was 47 days, range 36, 58) in the applied ELISAs and neutralizing antibodies during a follow up with serial measurements (Table S1).

### 3.3. Patient Care during SARS-CoV-2 Pandemic

**Figure 3:**
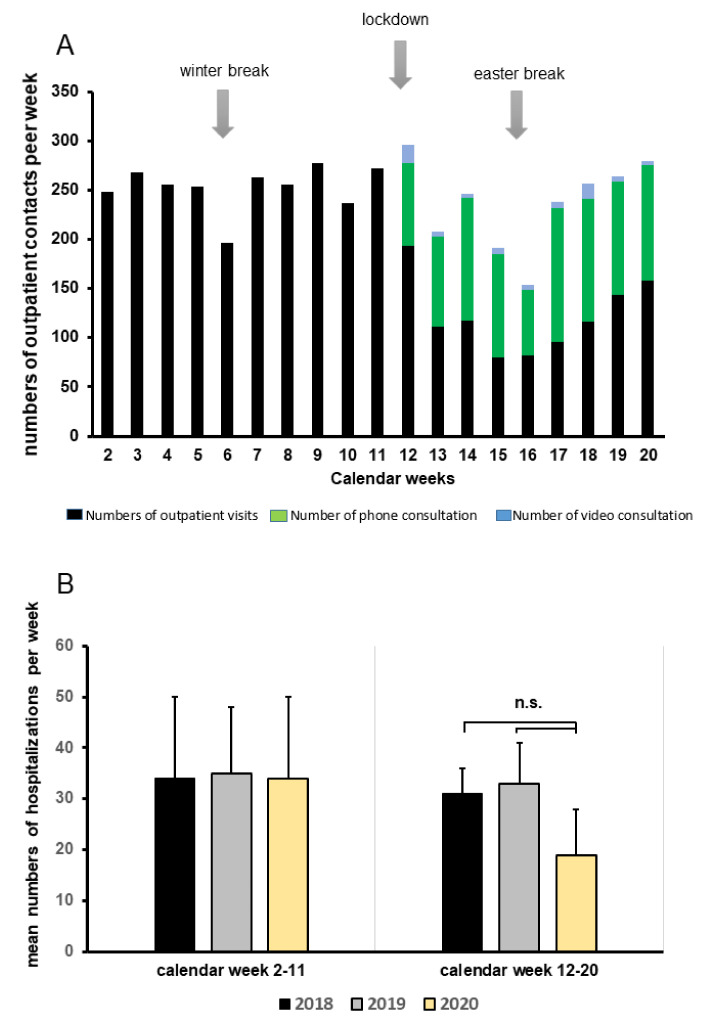
Patient care, alternative teleconsulting facilities, and hospitalization rates during the COVID-19 pandemic. (**A**) Number of outpatient contacts from January through May 2020 divided into outpatient visits, phone, or video consultations. (**B**) Mean numbers of weekly hospitalizations categorized into two time periods: calendar week 2–11 and 12–20 during 2018, 2019, and 2020 (yellow columns) compared to numbers in 2018 (black columns) and 2019 (grey columns); n.s. = not significant.

In 2019, 2044 patients after renal or combined renal transplantation came to our transplant outpatient clinic for follow up visits. After the onset of the COVID-19 pandemic, the numbers of scheduled patients were reduced to minimize risk of infection and virus transmission. To treat our patients properly, telemedicine was performed (e.g., per video, per calls) whenever indicated. As shown in Figure 3, numbers of patient visits in our outpatient section declined from January to May, while teleconsulting increased substantially from March until the end of May (Figure 3A). Importantly, compared with the last two years, we did not observe increased hospitalization rates of KTR, as depicted in Figure 3B.

We continued our deceased transplant program throughout the crisis, though we aimed to avoid high risk transplantations (KTR > 65 years, with severe comorbidities and high immunological risk with the need of more intense immunosuppressive induction therapy, or in cases of marginal donor organ quality). Within the first 2 months of 2020, we reported 30 transplantations (compared to 26 transplantations in 2019). From March to May 2020, this number fell to 24, compared to 44 in 2019. Moreover, we continued our regular living donation program until 18 March, followed by a four-week suspension before restarting on 22 April. Three planned live donations were postponed: one for a patient with vascular access problems and two for patients with AB0-incompatible grafts who had previously been treated with B-cell depletion by rituximab and received immunoadsorption. While we performed 11 live donations within the first two months in 2019 and 17 live donations from March to May, the numbers in 2020 were eight from January to February and 13 from March to May. To date, no COVID-19 infections in KTR or combined renal organ transplant patients have been reported after transplantation during the SARS-CoV-2 pandemic situation.

## 4. Discussion

The prevalence of COVID-19 in KTR with only mild symptoms is unknown. Serum screening for SARS-CoV-2-specific antibodies in our cohort revealed a serum prevalence of 7.2% (with mainly IgA antibodies). Using IFT to clarify specificity for SARS-CoV-2 infection, only 2 of 223 KTR (1%), both with reactive IgA and IgG ELISA test results, showed typical staining patterns. Both patients had previous common cold symptoms (26 and 135 days, respectively) before serum assessment. Thus, previous infection with SARS-CoV-2 remained possible. Beside these two patients, we assumed that the increased reactivity in the SARS-CoV-2 IgA ELISA in our cohort was caused by cross-reactive antibodies against common-cold viruses, rather than an undetected SARS-CoV-2 infection. Compared to the general population, seroprevalence studies for IgA are sparse or still ongoing. A German study investigated 217 frontline health-care professionals involved in COVID-19 patient care, wherein an IgG range of 1–2% and an IgA range of 4.1–4.6% were revealed [15]. Among public sector employees in Bremen (Germany), 6 out of 281 showed anti-SARS-CoV-2 IgG positive findings, while 31 were reported with solely IgA positivity [16]. Regarding respiratory symptoms as one of the main clinical manifestations of COVID-19, IgA antibodies have a higher sensitivity, while IgG antibodies are more durable, specific, and might be better suited for serosurveillance studies [17,18]. Remarkably, specific neutralizing antibodies were not detectable in the two KTR patients with potential SARS-CoV-2 infection. From recent reports, a lack of neutralizing antibodies in patients after mild infections or a fast disappearance is conceivable. Long et al. demonstrated that patients with or without mild symptoms of SARS-CoV-2 infection had weaker immune responses, either without the development of neutralizing antibodies or a fast reduction in IgG and neutralizing antibody levels [19]. Moreover, a SARS-CoV-2 serum prevalence of 0.97% was recently reported in a population-based study in another region of Germany (Nordrhein-Westfalen) that detected neutralizing antibodies in only one-third of tested persons with positive immunoassay results [20].

In this study, we showed robust immune responses in convalescent KTR after COVID-19 infection, detected by SARS-CoV-2 IgA antibodies against S1 and by IgG antibodies against S1 and N protein. The results were confirmed by IF and proof of neutralizing antibodies. Until now, IgA and IgG have remained positive in follow up serum tests, albeit with a decrease in ratio, which is paralleled by a decrease of neutralization antibodies. A decrease of IgG levels and neutralizing antibodies within 2–3 months after infection was observed by other researchers in a high proportion of individuals who recovered from SARS-CoV-2 infection [19,21]. We were able to demonstrate serum conversion after COVID-19 in all five KTR. Hartzell et al. reported serum conversion in 16 of 18 KTR after COVID-19 [22]. Benotmane et al. showed anti-SARS-CoV-2 IgM or IgG antibodies in all tested subjects (35 of 40) during the second week after symptom onset [23]. In spite of the fact that IFT and neutralization tests were missing in both studies to confirm SARS-CoV-2 specificity, the ability of immunosuppressed KTR to develop serum conversion could be demonstrated. Although validated serological assays are still needed, the high sensitivity and specificity of the SARS-CoV ELISA used in our study has already been reported in several other studies [10,17,24]. However, its impact on acquired immunity against SARS-CoV-2, especially in KTR, remains to be determined. Beyond serum responses, protective mechanisms such as SARS-CoV-2 specific T-cell responses have been discussed by others. Of note, two out of five transplanted patients with COVID-19 showed PCR-positivity of nose and throat swabs for several weeks and a putative delayed seroconversion. Our findings (and possible implications) are of special interest and need to be clarified in future studies.

Finally, the clinical impact of COVID-19 in kidney transplant recipients is still controversial. While some authors provide strong evidence for a deleterious outcome in KTR [25,26,27], mild courses were found without tremendously elevated mortality in other centers and countries [28,29,30]. In addition to age and certain preexisting comorbidities (e.g., obesity or lung disease), other factors that put KTR at risk for COVID-19 need to be determined. Given the relatively elective nature of renal transplant programs, the International Transplant Society (TTS) has recommended the temporary suspension of transplant programs in severely affected countries. However, we continued our deceased transplant program throughout the crisis and, except for a four-week interruption, continued our regular living donation program. The rationale to proceed with our transplant program was based on three major findings: first, as highlighted in our data and in line with the prevalence in the Berlin–Brandenburg region (around 17,000 cases/6.1 Million inhabitants (0.28%) at the end of our observation period), we found a very low prevalence of COVID-19 in KTR from our outpatient clinic (5 out of 2044, 0.24%). Second, the possibility of fast track testing for COVID-19 in cases of de novo transplantation allows us to provide a OVID-19 free pathway in terms of otherwise undiagnosed occult infections. To date, none of our recently grafted patients had tested positive during a follow up visit. In addition, according to the German organ procurement organization DSO (Deutsche Stiftung Organspende), all organ donors are tested for COVID-19 and only negative tested organ donors are allowed for donation [31]. Third, after the shutdown, we implemented effective containment strategies in our hospital, including face masks for source control, social distancing, hygienic education and doctor to patient distancing. In addition, we were able to provide a telemedical health system to all of our patients (MACSS). Such a system might be crucial to provide outpatient management of suspicious cases or mild forms of COVID-19. Comparing our transplant activities with those in other countries, we noticed that the majority of centers in Europe and the U.S. have stopped or dramatically reduced their transplant programs [32,33], which is reflected by the lower number of organ procurement activities [34]. However, one has to consider the benefits of early transplantation, especially in terms of living donation and transplant centers weighing risk versus benefits for the individual.

Our study was limited by the relatively small number of tested KTR and the lack of follow up serum measurements that investigated dynamical changes of the SARS-CoV-2 seroprevalence. The incident number of transplant patients with COVID-19 was only five, which impaired validation of serum responses.

## 5. Conclusions

Aside from diagnostic caveats, we believe that our screening approach could demonstrate a low rate of COVID-19 infections in our renal transplant cohort. In addition, we were able to provide novel insights with regards to antibody responses of SARS-CoV-2. Although our study was not able to provide rigorous data for numbers of occult infections, given the low number of positive screening results, we believe that the sum of implemented measures accounted, in a substantial way, for the fact that we were able to proceed with our transplant program.

## Figures and Tables

**Table 1:** Patient characteristics from outpatient section.

Characteristics	Recipients (*n* = 223)
Age at transplantation, years, median (IQR)	46 (34, 56)
Age at serum assessment in years, median (IQR)	54 (42, 64)
Type of transplant donor, *n* (%)	
Living donor	82 (36.8%)
Deceased donor	141 (63.2%)
Recipient gender male, *n* (%)	138 (61.9%)
Organ transplant, *n* (%)	
Kidney	210 (94.2%)
Kidney/pancreas	9 (64.0%)
Kidney/liver	3 (1.3%)
Multivisceral (kidney/pancreas/liver)	1 (0.4%)
Time from transplant to screening, months, median (IQR)	66 (14, 155)
Mean serum creatinine at screening, mg/dL (SD)	1.6 (0.9–3.1)

IQR = interquartile range; SD = standard deviation.

**Table 2:** The characteristics of five renal transplant recipient patients who experienced PCR-confirmed COVID-19.

No	Age, Years	Sex	Co- Morbi-Dities	Time since TX, Years	Baseline Crea, mg/dL	Symptom Start, Date	Date of Positive PCR	Crea, mg/dL	WBC G/L	CRP, mg/L	LDH, U/L	Date of Negative PCR	Time between PCR and 1st Serum Test, Days
1	78	M	ADPKD HTN CAD	6	1.6	March 9th	March 24th	3.1	3.5	68	676	April 24th	43
2	43	M	MPGN HTN	12	1.0	March 19th	March 30th	1.2	7.7	0.5	262	Apr 14th	31
3	53	M	HTN Obesity CHF COPD Diabetes	3	2.2	March 20th	April 1st	5.2	18.4	85	610	positive until April 20th	24
4	43	F	HTN HCV	15	1.4	March 23rd	April 1st	1.5	3.5	0.1	129	May 4th	43
5	61	M	Diabetes NPTX HTN	22	1.0	April 2nd	April 15th	1.0	5.0	31	229	May 19th	34

COVID-19 = Coronavirus Disease 2019, M = male, F = female, TX = transplantation, WBC = white blood cell, ADPKD = autosomal-dominant kidney disease, HTN = hypertension, CAD = coronary artery disease, MPGN = membrane-proliferative glomerulonephritis, CHF = chronic heart failure, COPD = chronic obstructive pulmonary disease, HCV = hepatitis C virus, NPTX = combined kidney-pancreas transplantation, Crea = Creatinine, CRP = C reactive protein, LDH = lactate dehydrogenase, PCR = polymerase chain reaction.

## Supplementary Materials

The following are available online at https://www.mdpi.com/2077-0383/9/11/3401/s1, Table S1: IgA and IgG ratios and interpretation of 5 PCR-confirmed COVID-19 patients in the KTR cohort.
