# Supplementary material for: Low Seroprevalence of SARS-CoV-2 Antibodies during Systematic Antibody Screening and Serum Responses in Patients after COVID-19 in a German Transplant Center
Source: J Clin Med. 2020 Oct 23;9(11):3401. doi: 10.3390/jcm9113401 (PMC7690804; doi:10.3390/jcm9113401)
Supplement: Supplementary file 1 [file jcm-09-03401-s001.pdf]

Journal of
Clinical Medicine

MDPI

Supplementary material

Supplementary Table S1. IgA and IgA ratios and interpretation of 5 PCR-confirmed COVID-19 patients in the KTR cohort. n.a. = not available (due to fatal outcome).

	week 1-3 post symptom onset			week 4-7 post symptom onset					week 18-24 post symptom onset				
**no**	**S1 IgA ratio**	**S1 IgG ratio**	**NCP IgG ratio**	**S1 IgA ratio**	**S1 IgG ratio**	**NCP IgG ratio**	**PRNT50**	**PRNT50**	**S1 IgA ratio**	**S1 IgG ratio**	**NCP IgG ratio**	**PRNT50**	**PRNT50**
1	0.46	0.15	0.24	8.83	6.73	5.25	>640	>640	6.07	8.3	1.38	>640	320
2	n.a.	n.a.	n.a.	11.35	5.71	1.64	80	20	2.42	0.95	0.87	<20	<20
3	1.02	0.22	0.76	>13	1.37	7.77	20	<20	n.a.	n.a.	n.a.	n.a.	n.a.
4	n.a.	n.a.	n.a.	2.37	1.42	0.81	320	80	1.25	1.82	1.11	40	<20
5	n.a.	n.a.	n.a.	>13	>13	4.84	>640	>640	11.12	5.97	3.69	640	160

S1= spike protein 1, NCP= nucleocapsid protein, PRNT50= plaque reduction neutralization test at dilution 50, PRNT90= plaque reduction neutralization test at dilution 90, IgA = Immunoglobulin A, IgG = Immunoglobulin G, COVID-19 = Coronavirus Disease 2019, KTR = kidney transplant recipient, ELISA = enzyme-linked immunosorbent assay, PCR = polymerase chain reaction.

.
